# A Case of Emergency Pericardial Drainage for Cardiac Tamponade Due to Dengue Fever

**DOI:** 10.7759/cureus.69712

**Published:** 2024-09-19

**Authors:** Kazuyuki Ishibashi, Mamika Motokawa, Mohammad Moniruzzaman, Aslam Ahmed, Shariful Islam

**Affiliations:** 1 Cardiovascular Surgery, Ship International Hospital, Dhaka, BGD

**Keywords:** acute pericardial effusion, cardiac tamponade, dengue virus infection, pericardial drainage, plasma leakage, severe dengue fever

## Abstract

Dengue fever is a febrile viral disease transmitted by mosquitoes. The symptoms of dengue fever are numerous and varied; however, cardiac tamponade is a particularly rare and atypical complication. This study describes a case of a 30-year-old male who presented with acute respiratory distress and chest pain on the ninth day after diagnosis of dengue fever. On admission, a computed tomography scan revealed a large pericardial effusion. The patient went into shock and was urgently treated with pericardial drainage, which resulted in rapid improvement of his symptoms. Emergent management of this rare but potentially fatal complication of dengue fever resulted in a favorable outcome in this case.

## Introduction

Dengue fever is the most widely distributed mosquito-borne viral infection in the world. However, it is difficult to determine the exact incidence of dengue due to the frequent occurrence of asymptomatic cases [1]. Typically, the early stages of the disease present with symptoms such as fever, nausea, headache, and osteoarticular pain. The fever usually resolves within two to seven days after onset, and symptoms begin to improve. However, a subset of patients may develop severe dengue, also known as dengue hemorrhagic fever or dengue shock syndrome, which can be fatal if not treated promptly and appropriately managed [2]. Currently, there are no specific antiviral drugs for dengue, and treatment is primarily symptomatic.

Severe dengue is characterized by plasma leakage, hemorrhagic complications, and multiple organ failure, often leading to life-threatening complications. One of the most serious complications associated with plasma leakage is pericardial effusion; however, cases of cardiac tamponade are extremely rare, with only a few reported worldwide.

In this study, we present an atypical case of severe dengue that presented with mild symptoms initially but rapidly progressed to cardiac tamponade, in which emergency drainage was performed with good results.

## Case presentation

A 30-year-old male with no previous medical history presented to our hospital with chest pain, dyspnea, and abdominal pain. Nine days before his visit, he was diagnosed with dengue fever after visiting his local doctor for fever, arthralgia, and orbital pain, but his symptoms were initially mild, and the patient was treated at home with antipyretics only after examination.

Three days before his hospitalization, despite an improvement in his general symptoms and resolution of his fever, he began to experience chest pain and reported persistent abdominal pain that began at the same time. When his respiratory distress suddenly worsened, he was urgently referred to our hospital.

On arrival, the patient was conscious and had no signs of skin rash, osteoarthralgia, myalgia, or bleeding tendency. His vital signs were as follows: temperature 37.3°C, heart rate 140 beats min, blood pressure 100/70 mmHg, and oxygen saturation (SpO_2_) 92%. His jugular veins distention were found, but auscultation revealed no heart valve murmurs and no pericardial friction rub. Laboratory tests showed a platelet count of 514,000/μL, WBC count of 21,880/μL, and hemoglobin of 12.5 g/dL, with troponin I and CKMB levels within normal limits. Inflammatory markers such as CRP and procalcitonin were a little elevated, while liver enzymes (aspartate aminotransferase {AST}, alanine aminotransferase {ALT}) were slightly above normal. Serum creatinine was normal. The serological examination was positive for dengue virus immunoglobulin M (IgM) and negative for immunoglobulin G (IgG). Blood cultures and serology for hepatitis B, hepatitis C, and HIV were all negative (Table 1).

**Table 1:** Laboratory findings at admission. AST: aspartate aminotransferase; ALT: alanine aminotransferase; CKMB: creatine kinase-MB

Laboratory test	Hospital admission	Normal range
Hemoglobin (g/dL)	12.5	10.0-14.0
Hematocrit (%)	36.9	36-46
Total leukocyte count (10^3^/µL)	21,880	4.0-11.0
Neutrophil (%)	84	40-75
Lymphocyte (%)	10	20-40
Monocyte (%)	5	2-10
Eosinophils (%)	1	1-6
Basophil (%)	0	<1
Platelets (10^3^/µL)	5,14,000	150-450
Creatinine (mg/dL)	0.98	0.52-1.04
AST (U/L)	71	14-36
ALT (U/L)	71	<35
Troponin I (ng/mL)	0.1	<0.16
CKMB (U/L)	8	<16
Procalcitonin (ng/mL)	33.65	<0.5
CRP (mg/L)	643	<10
Dengue IgM	Positive	Negative
Dengue IgG	Negative	Negative
Blood culture	Negative	Negative
HBs Ag	Negative	Negative
Hepatitis C Ab	Negative	Negative
HIV	Negative	Negative

**Figure 1:**
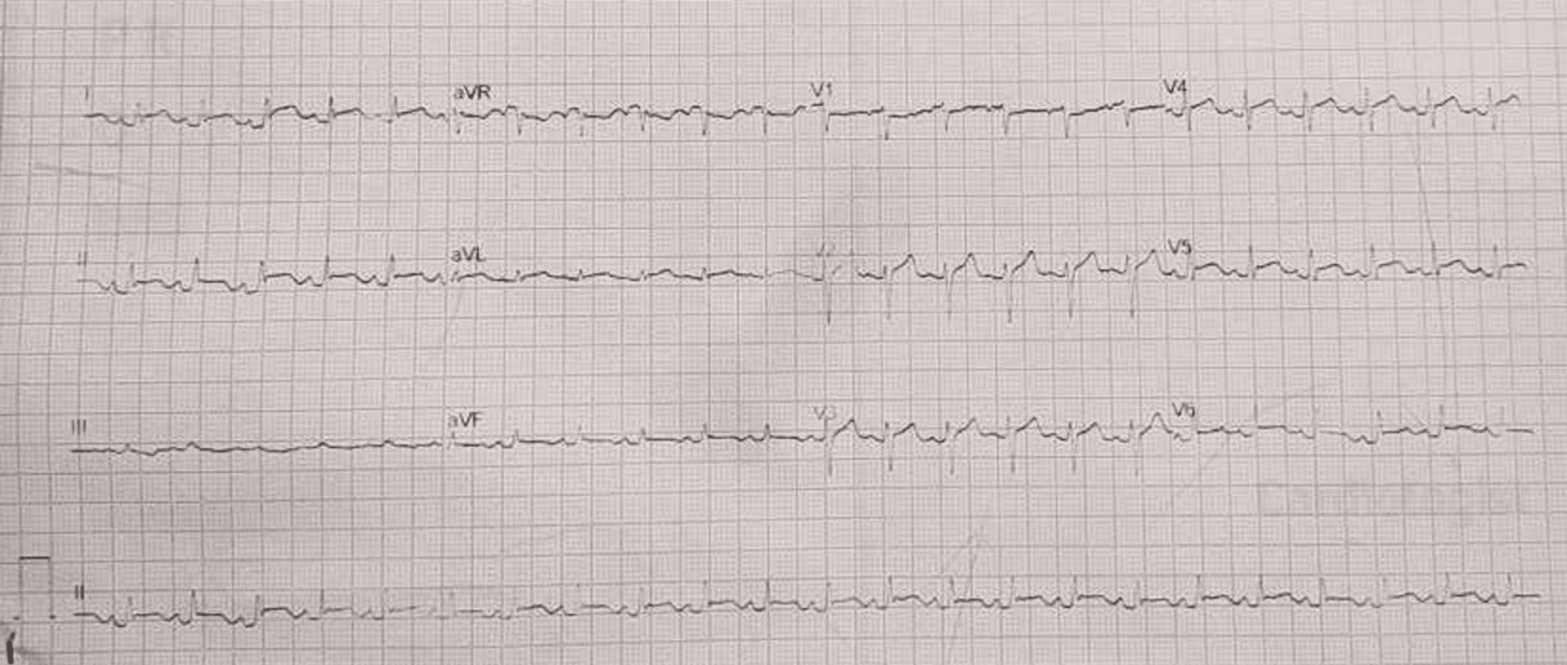
Electrocardiogram showed sinus tachycardia with low voltage QRS and slight ST-T elevations in I, II, V2-V6.

**Figure 2:**
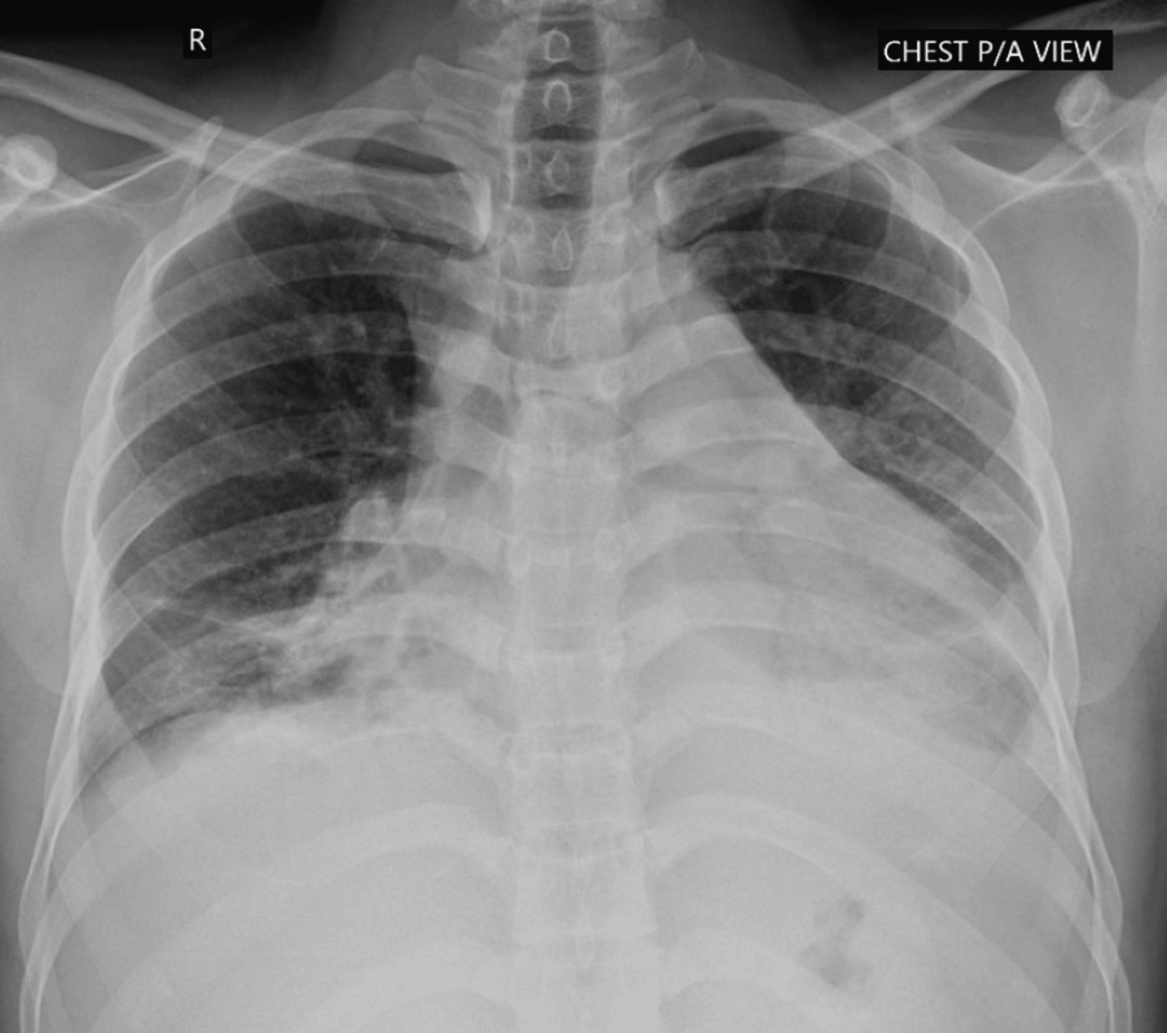
Chest X-ray at the time of admission showed enlargement of the cardiac shadow and a little bilateral pleural effusion.

An electrocardiogram showed sinus tachycardia with low voltage QRS and slight ST-T elevations in I, II, V2-V6 (Figure 1). Chest radiography revealed an enlarged cardiac shadow and a little bilateral pleural effusion (Figure 2). Echocardiography demonstrated a significant pericardial effusion with a preserved ejection fraction (EF) of 55%, without evidence of valvular abnormalities or regional wall motion abnormalities.

**Figure 3:**
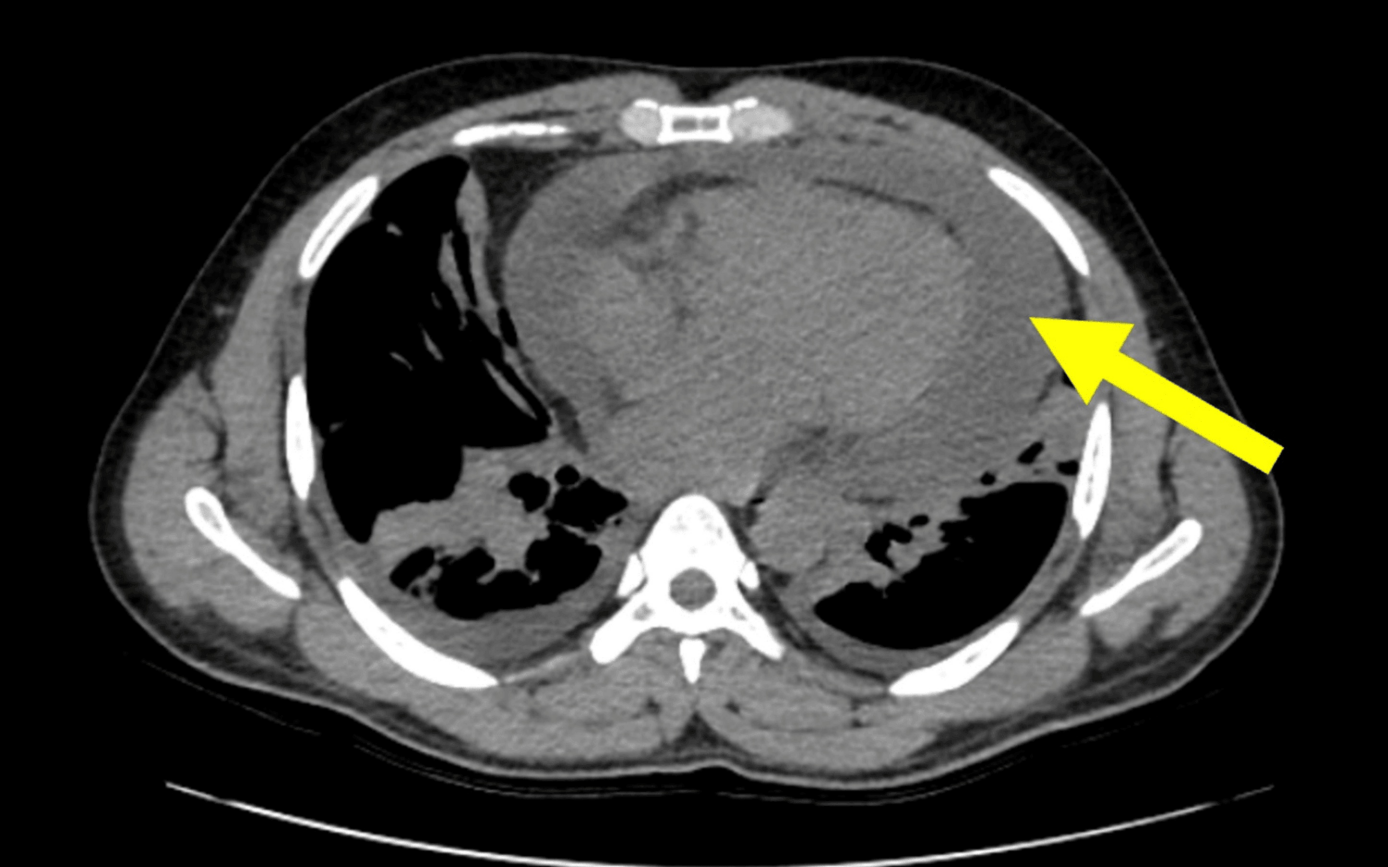
Computed tomography showed a large amount of pericardial effusion all around the heart.

Despite initial treatment, his respiratory status gradually worsened, and his SpO_2_ dropped to 90% while on supplemental oxygen. A chest computed tomography (CT) scan confirmed a large pericardial effusion (Figure 3).

Because of the patient’s deteriorating condition and systolic blood pressure in the 60 mmHg range, he underwent emergency pericardial drainage under general anesthesia, which removed 600 mL of yellowish fluid. Pericardiotomy was performed via subxiphoid approach. Following the procedure, the preoperative blood pressure was 60/30 mmHg but improved to 100/60 mmHg postoperatively, and his heart rate normalized to 90 beats per minute. The operation took one hour, and the patient was extubated immediately after surgery. The pericardial drain was removed 48 hours postoperatively due to significant clinical improvement. Analysis of the pericardial fluid revealed exudative effusion, with a WBC count of 1,800/mm^3^ (20% lymphocytes, 80% neutrophils), glucose of 6.9 mmol/L, lactate dehydrogenase (LDH) of 19 U/L, and protein of 10.6 g/dL. AFB stain, Gram stain, and bacterial cultures were negative (Table 2).

**Table 2:** Results for pericardial fluid characterization. AFB: acid-fast bacilli

Laboratory test	Result	Normal range
Total count of WBC (/µL)	1,800	4,500-11,000
Polymorph (neutrophils) (%)	80	40-70
Lymphocytes (%)	20	20-40
Pericardial fluid for AFB stain	AFB not found	Not found
Pericardial fluid for Gram stain	No organism is found	Not found
Bacterial culture	Negative	Negative
Pericardial fluid for protein (g/dL)	10.6	<1.5
Pericardial fluid for glucose (mmol/L)	6.9	>3.3

The patient was discharged six days after the pericardial drainage in good general condition, with complete symptomatic relief. Postoperatively, aspirin was started at 1,800 mg/day, decreased weekly, and discontinued after one month.

## Discussion

We present a case of mild dengue fever in a 30-year-old male who initially presented with only febrile symptoms and suddenly developed severe cardiac tamponade on the ninth day after his fever subsided. He had no other pre-existing medical conditions, and this was his first dengue infection, which is also very rare.

Dengue is a common febrile illness that is estimated to affect at least 390 million people worldwide each year [3]. Most patients recover spontaneously, but a small percentage of them develop a fatal condition called dengue hemorrhagic fever or dengue shock syndrome [1]. In 2009, WHO reclassified dengue fever into dengue fever (with or without warning) and severe dengue fever considering its different pathologies. According to this definition, the patient in this case initially had dengue fever (without warning), followed by the appearance of abdominal pain, one of the warning signs of dengue fever, seven days later, and a rapid change to severe dengue fever on the ninth day.

The main symptoms of severe dengue include plasma leakage, hemorrhagic lesions with thrombocytopenia, and multi-organ failure. However, there are few reports of pericardial effusion and cardiac tamponade as complications, although plasma leakage is one of the most serious factors [4]. In previous cases of dengue complicated by cardiac tamponade, there have been reports of patients with symptoms of severe forms of dengue fever from the onset despite primary infection, but in all these cases, comorbidities such as postpartum, hypothyroidism, and autoimmune diseases are thought to be risk factors in the severity of the disease [5-8]. Other cases of cardiac tamponade have been reported in cases due to secondary infection with dengue fever [9,10].

Like the present case, a case of primary dengue infection without pre-existing disease and concomitant cardiac tamponade has been observed to have persistent dengue-specific symptoms such as fever, thrombocytopenia, and rash, as well as warning signs such as fatigue and pleural effusion, followed by rapid accumulation of pericardial fluid [11]. In the present case, there was no thrombocytopenia or rash, and rapid pericardial effusion occurred within a few days after resolution of fever, with abdominal pain as the only symptom initially suggestive of dengue fever (with warning signs). Other reports have shown that the transition from symptoms of dengue fever (with warning) to severe dengue fever is very rapid, so it is important not to miss these atypical common symptoms of dengue fever to save lives.

Plasma leakage is a hallmark of severe dengue and an important factor in the development of pericardial effusion. The high protein content of the pericardial fluid component indicates that it is exudative in this case. The mechanism leading to pericardial tamponade is still unknown, but the cause is that dengue virus infection damages vascular endothelial cells, increasing vascular permeability and causing plasma components to leak from blood vessels, resulting in exudate. The inflammatory response to dengue infection is accompanied by the release of prostaglandins and cytokines, which further increase vascular permeability and promote plasma leakage. The thrombocytopenia and coagulopathy caused by dengue virus may cause vascular damage and promote further plasma leakage. In general, plasma leakage persists for about 48 hours after resolution of fever, followed by rapid recovery [12]. Understanding these mechanisms is essential for managing plasma leakage in dengue patients and for understanding serious complications such as cardiac tamponade.

On the other hand, dengue virus has four serotypes (DENV-1 to DENV-4), and secondary infection with different serotypes may cause more severe symptoms due to antibody-dependent enhancement (ADE) [13]. In the present case, IgG was negative but IgM of DENV was positive, suggesting primary infection with DENV. Although serotyping was not performed in this case, the patient was severely ill despite the primary infection. Some reports have suggested that certain serotypes, such as DENV-2, are associated with cardiac-related complications. It is now known that not only serotypes but also various factors such as age, gender, nutritional status, autoimmune response, host genetic factors, ethnicity, medical history, viral load, and virulence play a role [13,14].

Dengue fever is endemic in many parts of the world and is a disease that often resolves spontaneously. However, some patients develop severe dengue fever after the fever resolves. Currently, there are no markers that can predict the transition from dengue fever to severe dengue fever, only common subjective symptoms such as nausea and abdominal pain, which are warning signs. Therefore, understanding the atypical symptoms and course of dengue patients will lead to early diagnosis and timely treatment, preventing serious complications and providing good patient outcomes.

## Conclusions

Emergency pericardial drainage was successfully performed in a patient who presented with shock, sudden dyspnea, and chest pain nine days after being diagnosed with mild dengue fever. This case highlights the importance of clinicians being aware of the rare, atypical, and potentially fatal complications associated with dengue fever, a common infectious disease. Even when dengue fever initially presents as mild, serious complications such as plasma leakage, hemorrhagic symptoms, and multi-organ failure often develop after the patient appears to have recovered, making close monitoring and attention to warning signs extremely important.
